# Mapping the Convergence of Frontier Technologies for Major Environmental Challenges: A Chemical and Molecular Perspective on the Use of AI for Climate Action and Antimicrobial Resistance

**DOI:** 10.3390/molecules31101571

**Published:** 2026-05-08

**Authors:** Segundo Jonathan Rojas-Flores, Rafael Liza, Renny Nazario-Naveda, Félix Díaz, Daniel Delfin-Narciso, Moisés Gallozzo Cardenas, Luis Cabanillas-Chirinos

**Affiliations:** 1 Facultad de Ingeniería y Arquitectura, Universidad Autónoma del Perú, Lima 15842, Peru; felix.diaz@autonoma.pe; 2 Escuela de Posgrado, Universidad Continental, Lima 15113, Peru; rlizan@continental.edu.pe; 3 Departamento de Ciencias, Universidad Tecnológica del Perú, Trujillo 13011, Peru; c30216@utp.edu.pe (R.N.-N.); c21228@utp.edu.pe (M.G.C.); 4 Grupo de Investigación en Ciencias Aplicadas y Nuevas Tecnologías, Universidad Privada del Norte, Trujillo 13011, Peru; daniel.delfin@upn.edu.pe; 5 Institutos y Centro de Investigación, Universidad Cesar Vallejo, Trujillo 13001, Peru; lcabanillas@ucv.edu.pe

**Keywords:** artificial intelligence, antimicrobial resistance, climate change, metagenomics

## Abstract

The planet faces the critical interconnected challenges of climate change and antimicrobial resistance (AMR); these two crises mutually reinforce each other, threatening global health and ecosystem stability. This study conducts a systematic documentary analysis to map the convergence and identify the structural gaps between two key technological domains: artificial intelligence (AI) for climate action and molecular methods for AMR. The methodology was based on a corpus of 179 scientific documents indexed in Scopus (2010–2025), analyzed with data science tools to identify trends, collaborations, and impact. Quantitative results revealed clear leadership by the United States, accounting for 37.4% of publications, followed by China (26.8%); this leadership reflects the concentration of high-throughput molecular surveillance infrastructure and data science clusters essential for monitoring the environmental resistome. In terms of scientific impact, Spain showed the highest average, with 32.8 citations per article. The most influential work, a review on food security and sustainability, accumulated 275 citations. Network analysis identified authors such as Zhu, Yongguan, with 240 citations in total, as central nodes in international collaborations. Thematically, metagenomics and machine learning emerged as mature and interconnected research cores. This analysis confirms a solid yet still fragmented relationship between the two fields. The analysis reveals that, while metagenomic tools dominate the current literature, a gap persists in correlating genotypic resistance potential with functional phenotypic expression under changing climatic stressors. The results confirm a solid yet still fragmented foundation, highlighting the need for hybrid platforms that transition from descriptive bibliometrics to functional integration for designing systemic solutions. Future work should prioritize the development of hybrid platforms, such as intelligent biosensors, and collaborative governance frameworks that accelerate effective responses to these dual crises.

## 1. Introduction

The planet faces an unprecedented environmental crossroads, defined by two interconnected crises that threaten global health and ecosystem stability [1]. On the one hand, climate change is advancing with alarming severity. According to the Intergovernmental Panel on Climate Change (IPCC, 2023), human activities have caused global warming of approximately 1.1 °C above pre-industrial levels, with projections under current emission scenarios indicating that temperatures could reach or exceed 1.5 °C within the next two decades [2,3]. This increase translates into extreme phenomena: the World Meteorological Organization reported that the period of 2015–2024 was the warmest decade on record, marked by events such as floods, megadroughts, and heatwaves that displaced millions of people and caused trillions in economic losses [4].

In parallel, antimicrobial resistance (AMR) has become a silent pandemic [5]. The Lancet (2024) stated that infections caused by resistant bacteria were directly associated with 1.27 million deaths worldwide in 2023, surpassing mortality figures for HIV/AIDS and malaria [6]. The World Health Organization (WHO) warns that, without decisive action, this figure could rise to 10 million annually by 2050, with a cumulative cost to the global economy of up to USD 100 trillion [7]. The dissemination of resistance genes in the environment, driven by the improper discharge of pharmaceutical, agricultural, and hospital effluents, creates a vicious cycle in which environmental pollution exacerbates public health crises [8].

The relevance of addressing these challenges in an integrated manner lies in their profound interconnection and planetary scale of impact. Climate change is not merely an ecological disruptor; it acts as a threat multiplier for AMR [9,10]. Rising temperatures can accelerate bacterial mutation rates and horizontal gene transfer, while phenomena such as flooding facilitate the spread of resistant pathogens from contaminated sources into water and soil [11,12]. Conversely, the production of antimicrobial agents and the management of associated waste contribute to greenhouse gas emissions [13], making isolated approaches insufficient. The importance transcends environmental and health dimensions, extending to economic and food security concerns. For instance, agriculture—a vital sector for humanity—is simultaneously vulnerable to climate stress and a major user of antimicrobials (thus contributing to AMR) [14]. In parallel, AMR has become a silent and multifactorial public health problem. According to the WHO, the inappropriate and excessive use of antimicrobials in human medicine, veterinary practice, and agriculture constitutes the primary driver of resistant pathogen emergence. In hospitals and clinics, overprescription, self-medication, and unregulated access to antibiotics generate selective pressure that favors the survival of resistant strains (CDC) [15].

The molecular mechanisms underlying AMR include point mutations in target genes and horizontal gene transfer via plasmids, transposons, and integrons, as well as the formation of biofilms, which confer tolerance and facilitate genetic exchange. These adaptations enable bacteria to survive even in the presence of antibiotics, complicating the treatment of common infections [16,17]. The public health implications are profound: untreatable infections, such as those caused by Methicillin-resistant Staphylococcus aureus (MRSA) or carbapenem-resistant Enterobacteriaceae, increase mortality and morbidity rates; the pressure on healthcare systems; and the risk of setbacks in routine medical procedures such as surgery, transplantation, and chemotherapy. In the United States, for example, more than 2.8 million resistant infections are estimated to occur annually (CDC). Globally, AMR was directly responsible for 1.27 million deaths in 2019 and contributed to 4.95 million [18]. This health crisis is intertwined with climate change: rising temperatures accelerate mutations and genetic transfer, while extreme events such as flooding facilitate the spread of resistant pathogens. Addressing AMR and climate change in an integrated manner—through artificial intelligence and molecular methods—thus represents a strategic priority for global resilience [19].

Given this complex and dynamic landscape, a critical and visually compelling synthesis of existing knowledge is required. A systematic review or bibliometric analysis based on the Scopus database offers a distinctive advantage [20]. Scopus is recognized for its broad coverage of peer-reviewed scientific literature and high-quality indexing, surpassing other databases in metadata comprehensiveness and interdisciplinary reach—crucial for a topic spanning environmental sciences, computing, biochemistry, and public health [21]. Employing tools such as R Studio for statistical analysis and bibliographic data manipulation, VOSviewer for mapping co-citation and keyword co-occurrence networks, and Plotly (v 0.0.73) for generating interactive visualizations of temporal trends and publication impact transforms a traditional review into an advanced meta-analytical study [22,23]. Despite progress, a gap persists in the systematic integration of climate-focused AI and molecular methods for AMR within a unified analytical framework. This gap limits the understanding of environment–health interactions, and addressing it is crucial for designing more effective interdisciplinary strategies.

The general objective of this research is to conduct a documentary analysis of the integration of frontier technologies—AI for climate action and molecular methods for AMR—as a response to the most pressing environmental and health challenges. Specifically, this study will: Q1: identify publication trends in AI applied to climate science; Q2: map scientific collaboration networks in the application of molecular methods in AMR studies; Q3: compare citation evolution and impact factors across both fields; Q4: visualize thematic patterns; and Q5: propose emerging research lines that integrate both approaches. This documentary analysis seeks to build bridges between artificial intelligence applied to climate action and molecular methods addressing antimicrobial resistance. By integrating these frontier technologies, this study aims to provide robust evidence and interdisciplinary perspectives that strengthen research, policy formulation, and response capacity to the most urgent environmental and health challenges. Given the centrality of chemical processes in both climate change (e.g., greenhouse gas emissions and atmospheric chemistry) and antimicrobial resistance (e.g., drug design and environmental fate of antibiotics), this review places a special emphasis on the chemical and molecular dimensions of the frontier technologies under analysis. Despite advances in both fields, a gap persists in the systematic integration of climate-applied AI and molecular methods targeting AMR within a unified analytical framework. The present study addresses this gap through a documentary analysis that not only maps scientific production but also extracts thematic patterns, collaboration networks, and emerging trends, providing a roadmap for future interdisciplinary research.

## 2. Results and Analysis

Table 1 presents a documentary analysis of the ten most influential and highly cited scientific articles published between 2010 and 2025 at the intersection of artificial intelligence (AI), sustainability, and antimicrobial resistance (AMR). These articles are distinguished by their interdisciplinary approach, integrating chemical, molecular, and computational dimensions to address global environmental challenges. The ranking is led by the work of Vågholm et al. (2020) [24], which has accumulated 275 citations and examines food security and sustainability within the framework of the Sustainable Development Goals. It is followed by foundational research on the microbiome and genomic surveillance [24], such as that by Zhang et al. (2021), who examined soil contaminants, and that by Arango-Argoty et al. (2019), who developed the NanoARG platform for the detection of resistance genes through metagenomics [25,26]. The analysis of these documents reveals a clear trend: while studies on AI and sustainability demonstrate greater maturity and visibility, research on AMR and molecular methods reflects a more recent evolution characterized by highly specialized technical methodologies. Taken together, the table illustrates how tools such as machine learning and data analytics have become essential for interpreting the complexity of the environmental resistome and phenotypic expression under climate stressors.

Table 2 profiles the leading authors applying AI in climate studies and molecular methods in antimicrobial resistance studies (2010–2025). The researchers are predominantly affiliated with institutions in China, Australia, Spain, and the U.S. Zhu, Yongguan, leads in total citations (240), followed by Zhang, Qi (185), and Zhang, Zhenyan (172). High co-authorship counts (7 to 29) reflect strong transnational and interdisciplinary collaboration. However, “Unknown” affiliations—such as for Qian, Haifeng—suggest indexing limitations or outdated institutional data in Scopus. When comparing these results with those of recent studies published between 2023 and 2026, significant continuities and divergences emerge. On the one hand, the consolidation of China and the United States as hubs of scientific production in environmental AI and AMR aligns with the findings reported by Gupta and Bhandary (2024) [12] and Nayak et al. (2023) [14], who highlight Asian and North American leadership in applying AI for resistant pathogen surveillance. However, while Table 2 reflects strong Spanish representation through CREAF and UAB, more recent reviews such as Kumari and Saraogi (2025) emphasize the growing role of India and South Korea in developing high-throughput molecular platforms, suggesting an expanding scientific geography [19].

**Figure 1:**
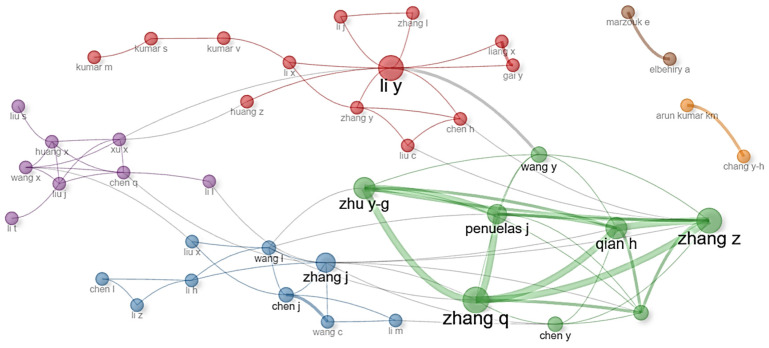
Collaboration networks among authors applying AI in climate studies and molecular methods in AMR studies (2010–2025). Nodes represent authors; edges represent co-authorship relationships. Node size reflects total link strength; link thickness indicates co-authorship frequency. Colors denote clusters identified by the VOS clustering algorithm (resolution = 1.0). See Section 5.3 for detailed parameters.

Figure 1 illustrates a scientific collaboration network among authors applying artificial intelligence to address climate challenges and molecular methods to address antimicrobial resistance (AMR). Each node in the figure represents an author, and the connections between them indicate co-authorship relationships. Node size reflects the centrality or influence of the author within the network, while link thickness indicates the intensity of the collaboration. Colors group authors into thematic or institutional clusters, revealing scientific communities with shared interests. Prominent authors include Li Y., Zhang Z., Zhang Q., Qian H., Zhu Y.-G., and Peñuelas J., who demonstrate not only high scientific productivity but also strong interdisciplinary articulation. For example, Zhu Y.-G. and Peñuelas J. appear as key nodes in networks linking environmental microbiology with sustainability, while Zhang Q. and Qian H. are situated in clusters integrating biotechnology and ecosystem analysis.

**Figure 2:**
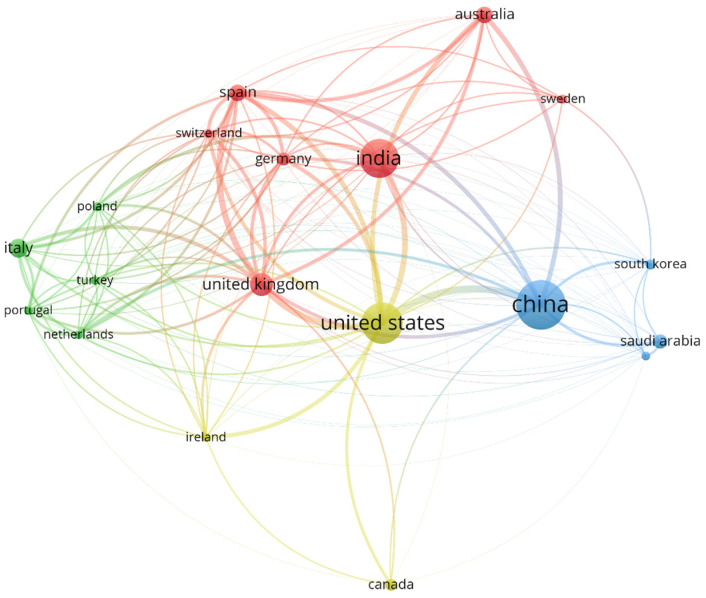
International collaboration networks in the application of AI in climate studies and molecular methods in AMR studies (2010–2025). Nodes represent countries; edges represent co-authorship collaborations. Node size is proportional to the number of publications; link thickness reflects collaboration intensity. Colors indicate clusters identified by the VOS algorithm. See Section 5.4 for detailed parameters.

Figure 2 illustrates international scientific collaboration networks in the application of artificial intelligence in climate studies and molecular methods in antimicrobial resistance (AMR) studies. This visualization reflects a predominantly transnational cooperation structure, with key nodes located in China, the United States, Spain, and Australia, confirming the globalized nature of research in these disciplines. The strongest and most frequent links are observed among institutions such as the Institute of Urban Environment (China), the Universitat Autònoma de Barcelona (Spain), and the Virginia Tech College of Engineering (USA), evidencing strategic alliances that have facilitated knowledge transfer and co-authorship in high-impact publications. The density of connections suggests a highly interconnected scientific community, where collaboration among microbiologists, data scientists, and environmental engineers is more the norm than the exception.

Table 3 presents a comparative analysis of the scientific output and impact by country. The data reveal the leadership of the United States in publication volume (67) and the total number of citations (1116), while the United Kingdom and Spain stand out for the highest impact per article (with 26.96 and 32.8 citations on average, respectively). China ranks second in production, followed by India, although the latter shows a lower impact. The funding column reflects a correlation between institutional support and productivity, particularly in the USA, the UK, and Spain, which also show the highest number of funded papers.

Table 4 presents a quantitative analysis of the most productive and impactful scientific journals in research integrating antimicrobial resistance (AMR), environmental microbiology, and sustainability between 2010 and 2025. The data reveal a competitive landscape led by three journals with eight articles each: Antibiotics, Frontiers in Microbiology, and Science of the Total Environment. However, the impact metrics show significant differences. Science of the Total Environment stands out with the highest total number of citations (TC = 210) and a solid h-index of 6, suggesting sustained influence and high visibility within the environmental science community. Frontiers in Microbiology, despite its high productivity, presents a relatively low m-index (0.571) and an h-index of 4, indicating more recent output or a citation distribution less concentrated in highly influential articles. Antibiotics, with a high m-index (1.5), reflects a very recent and pronounced impact, likely driven by the urgent global agenda on AMR. Specialized journals such as Water Research and Microbiome show a high-impact profile disproportionate to their article volume, with 206 and 188 citations in total, respectively, confirming their central role in key thematic niches. The dominance of journals such as Water Research, Science of the Total Environment, and Environmental Science & Technology is not coincidental. It reflects that the field has an intrinsically interdisciplinary nature but remains anchored in environmental sciences. Water Research has established itself as the reference outlet for studies with direct implications for process engineering and wastewater operations, which explains its leadership in articles. Environmental Science & Technology tends to publish work with a deeper mechanistic and chemical emphasis, making it the ideal venue for studies integrating proteomics and polymer chemistry [1,16]. Meanwhile, the high productivity of Science of the Total Environment reflects its broad scope and role as a platform for large-scale monitoring, occurrence, and review studies. This stratification across journals not only indexes productivity but also delineates the field’s “disciplinary frontiers”: more applied and engineering-oriented studies find their home in water-focused journals, while mechanistic and environmental chemistry studies are found in journals with a broader science and technology orientation. Understanding this structure is essential for authors seeking to position their work within the appropriate niche.

**Figure 3:**
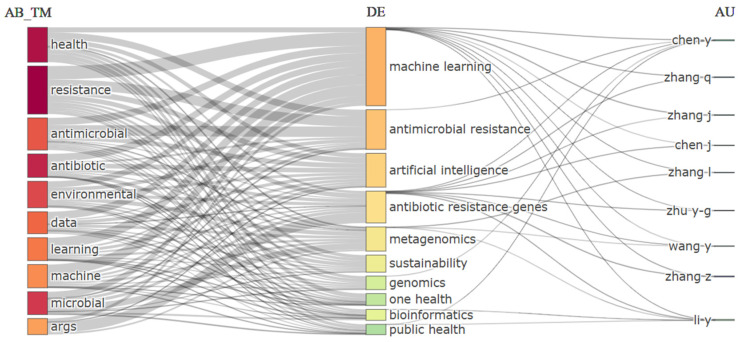
Importance matrix of the main concepts of the study (AI, AMR, and environment) across different sections of the analyzed documents. The matrix integrates terms from abstracts/titles (AB_TM, yellow), author keywords (DE, red), and author names (AU, green). Point size is scaled by the importance score (frequency × centrality). See Section 5.5 for detailed parameters.

Figure 3, showing an importance matrix of concepts, breaks down the thematic cores and key actors at the intersection of AI, AMR, and environmental sciences according to their presence in abstracts/titles (AB_TM), descriptors (DE), and authors (AU). At the fundamental lexical level (AB_TM), generic but essential terms such as health, resistance, antimicrobial, antibiotic, and environmental stand out, confirming that the documentary corpus is built upon the conceptual triangle of health–environment–resistance. The presence of data, learning, and machine evidences the transversal penetration of data science into research. At the level of specialized descriptors (DE), integrative and methodological concepts strongly emerge, including machine learning, artificial intelligence, metagenomics, genomics, bioinformatics, and frameworks such as One Health and public health, signaling the analytical sophistication and systemic orientation that characterize more applied literature. The author cloud (AU) is dominated by researchers of Chinese origin (prefixes Chen-, Zhang-, Zhu-, Wang-, and Li-), reflecting China’s already observed leadership in scientific production in this field, particularly in environmental microbiology and data analysis. The institutional collaboration map presents a clustering coefficient (Q = 0.58), indicating clear specialization. Chinese institutions (Tongji University, SKLPC) form a cluster with the highest total link strength (e.g., 120), dominating research output. The University of Technology Sydney (Australia) emerges as a node with high eigenvector centrality, reflecting strong connections with other influential nodes and serving as a bridge to the Asia-Pacific region. The presence of the Universitat Politècnica de València, with a link strength of 8 to the Chinese cluster, quantifies Spanish–Chinese collaboration in this field. This network topology, characterized by a dominant hub with international spokes, suggests a typical “center–periphery” collaboration model found in technological domains, where a single nation (China) has made a coordinated, large-scale investment.

**Figure 4:**
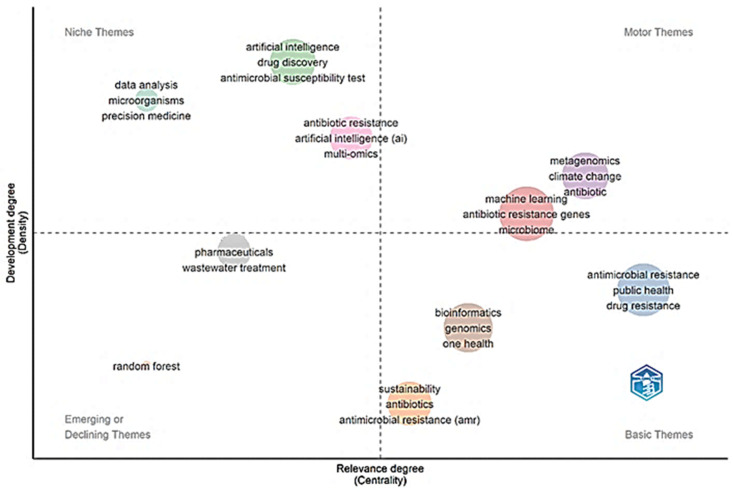
Strategic map of topics by centrality (relevance) and density (development) in research on artificial intelligence, antimicrobial resistance, and environmental sustainability. Quadrants: upper right = motor themes; lower right = basic themes; upper left = niche themes; lower left = emerging/declining themes. Point size indicates the number of keywords per cluster. See Section 5.6 for detailed parameters.

Figure 4 presents the degree of relevance and interconnection of topics within the knowledge network, as well as their internal development and cohesion. The arrangement of topics into the categories “Niche Topics,” “Emerging or Declining Topics,” and “Basic Topics” reveals the intellectual structure of the domain. A significant concentration is observed in the “Niche Topics” category, where concepts such as precision medicine, drug discovery, multi-omics, metagenomics, machine learning, antibiotic resistance genes, and wastewater treatment show maximum values in both centrality and density (1.0). This indicates that they constitute highly specialized research cores, are well developed internally, and act as crucial bridges connecting subfields such as bioinformatics with public health or environmental engineering with genomics. The presence of climate change in this group reinforces the central analysis of the document regarding the interconnection of the climate and health crises. By contrast, the categories of “Basic Topics” and “Emerging or Declining Topics” appear with more generic values, which may suggest a phase of consolidation or transition for more fundamental or novel themes that have not yet reached a defined structure within the analyzed network.

## 3. Discussion

In Table 1, it can be observed that articles on AI applied to environmental monitoring and sustainability stand out for their interdisciplinary approach. Gupta et al. (2021) explore how data analysis can transform research in environmental science and engineering, opening new possibilities for managing complex information [28]. Moreno-Indias et al. analyze the challenges of machine learning in human microbiome studies, underscoring the need for robust techniques to process highly complex datasets [30]. Mahfuz et al. investigate the implementation of smart technologies in modern pig farming, linking global demand for animal protein with sustainable practices [33]. Decouttere et al. occupy an intermediate category, connecting immunization with the Sustainable Development Goals and showing how health interventions can generate multisectoral impacts beyond healthcare [29]. Although not directly focused on AI or AMR, its inclusion contributes a holistic perspective on sustainability and public health. By comparing the groups, it is found that articles on AI and sustainability (Vågholm et al. [24]; Gupta et al. [28]; Mahfuz et al. [33]) have a higher number of citations, indicating greater maturity and visibility within the scientific community. In contrast, studies on AMR and the microbiome (Zhang et al. [25]; Arango-Argoty et al. [26]; Chen et al. [27]; Liu et al. [31]; Buelow et al. [32]) reflect a more recent evolution, with specialized approaches and advanced molecular methodologies.

Likewise, Vågholm et al. (2020) lead with 275 citations, analyzing food security and sustainability within the SDG framework [24]. Zhang et al. (2021) examine Gammaproteobacteria and soil contaminants [25], while Arango-Argoty et al. (2019) introduce NanoARG, a metagenomic platform for resistance gene surveillance [26]. Chen et al. (2019) track AMR genes in rivers using crAssphage markers [27]; Liu et al. (2021) explore the co-selection of antibiotic and metal resistance in agroecosystems [31]; and Buelow et al. (2021) assess hospital-derived contaminants in urban sanitation systems [32]. It is important to note that studies on the molecular detection of resistance genes—such as those by Arango-Argoty et al. [26], Chen et al. [27], and Liu et al. [31]—focus on genotypic resistance potential, i.e., the presence of genetic determinants conferring resistance traits. However, the presence of these genes does not always translate into expressed phenotypic resistance due to factors such as gene regulation, differential expression, or environmental conditions. Conversely, phenotypic resistance can occur in the absence of known resistance genes, mediated by mechanisms such as persister tolerance or biofilm formation. This distinction is essential for accurately interpreting the scope and limitations of the molecular methods examined in this study.

The convergence between both domains remains incipient, but works such as the study on NanoARG [26] and that on organic fertilization [31] demonstrate that hybrid platforms integrating AI, metagenomics, and environmental analysis are already emerging. In terms of impact metrics, the authors in Table 2 show a high average number of citations per article (between 45.5 and 88), indicating sustained influence in the literature. This contrasts with recent studies that, although citing these same authors, place greater emphasis on emerging tools such as CRISPR, advanced optical sensors, and federated learning models, as described by Taha et al. (2024) [2] and Olatunji et al. (2024) [34]. These newer works tend to be more specific in technological applications, whereas the authors in Table 2 represent a broader and more foundational base in environmental microbiology and data science. Integrating proteomics with metagenomics would allow correlations between changes in protein expression (such as those reported by Sharma et al. [3]) and shifts in the abundance of antibiotic resistance genes (ARGs) within the microbial community.

Figure 1 reveals a highly connected collaborative structure, with several authors bridging clusters, suggesting an increasingly transdisciplinary science. Studies such as those by Pandey et al. (2025) [18] and Taha et al. (2024) [2] introduce new actors from India, South Korea, and West Africa, who do not yet appear as central nodes in Figure 2 but are emerging in high-impact publications. Furthermore, current networks increasingly incorporate not only co-authorship but also collaboration through open-source code, data repositories, and environmental surveillance platforms [35], as evidenced in the NanoARG and AI-integrated biosensors described by Lawal et al. (2025) [1]. Figure 2 thus provides a robust snapshot of the scientific collaboration in frontier technologies while also highlighting the need to update bibliometric maps to include new forms of digital cooperation and emerging actors. The science of the future will be measured not only by citations or co-authorships but also by its ability to integrate knowledge, share resources, and collectively respond to global challenges such as climate change and AMR [36,37]. The hegemony of the United States and China identified in the analysis is not merely numerical; it represents a centralization of biotechnological infrastructure (e.g., NanoARG platforms) that effectively dictates the global agenda for molecular research [38]. This indicates a trend toward more hybrid research ecosystems, where the triple helix (academia–industry–government) is gaining increasing relevance. Another differentiating aspect is the methodological focus of recent collaborations [39]. While the network in Figure 2 reflects projects based on metagenomics, climate modeling, and traditional bioinformatics analysis, publications from 2025 to 2026 such as the study by Pandey emphasize the integration of federated data, explainable AI (XAI) models, and real-time IoT sensors, requiring more technical and specialized collaborations [40]. This has led to the formation of international consortia focused on data standards, AI ethics, and reproducibility—dimensions still incipient in the 2010–2025 period [41,42]. Thus, Figure 2 represents the historical collaborative foundation that enabled significant advances at the intersection of climate AI and AMR. However, a comparison with more recent literature reveals a transition toward more distributed, inclusive, and technologically advanced networks, with new actors, cooperation formats, and research priorities oriented toward the practical implementation and global governance of these frontier technologies [41,43].

When comparing the results of Table 3 with those of the most recent literature, emerging trends not captured in the table become evident. For example, Ali et al. (2023) highlight the rise of scientific output in Southeast Asian countries, particularly Indonesia and Malaysia, in AI applied to environmental sustainability, supported by national open science policies and South–South cooperation [11]. Likewise, Mee et al. (2025) note that research in convergent technologies (AI, biotechnology, and nanoscience) is attracting impact investment and venture capital, potentially reshaping the traditional funding landscape dominated by public agencies [38]. Regarding publication quality and influence, Khan et al. (2025) [17] caution that metrics such as the average number of citations may vary significantly depending on the discipline and language of publication, which could explain the differences observed between India and Europe. Furthermore, AMRos Cordeiro et al. (2024) emphasize the growing importance of public–private consortia in AMR research, particularly in Germany and Canada, countries that already show high impact in Table 3 (with 21.5 and 20 citations on average, respectively) [23]. Studies such as Ahlawat et al. (2024) highlighted the role of citizen science platforms and open data repositories in expanding the reach of research, enabling participation from countries with a more limited scientific infrastructure [15]. This suggests that, in the future, the geography of collaboration and impact may become more distributed and inclusive, transcending the centralized patterns reflected in Table 3. Studies (Table 4) such as those by Karnwal et al. (2025) [39] and Oladipo et al. (2025) [40] have identified explosive growth in interdisciplinary and open-access journals that merge artificial intelligence, data science, and molecular biology, including ACS Synthetic Biology and Biosensors and Bioelectronics. Although these journals contributed fewer articles to the corpus analyzed up to 2025, they are rapidly gaining traction by publishing research on CRISPR tools, nanomaterial-based sensors, and AI-driven diagnostic platforms for AMR surveillance [39,40].

Likewise, reviews such as Guo et al. (2025) highlight an increase in applied and translational science studies in high-impact journals such as Nature Communications and Advanced Drug Delivery Reviews, focused on convergent technological solutions [41]. This shift suggests a transition from fundamental research in environmental microbiology—dominant in journals such as Environmental Pollution and Chemosphere—toward more applied science oriented to intervention and technological innovation. Thus, while Table 4 reflects the consolidated foundation of research, the current frontier of knowledge is being reconfigured toward journals that prioritize technological integration, open science, and transdisciplinary approaches to address AMR and environmental sustainability in a synergistic manner. When contrasted with the broader literature, emerging emphases and new layers of complexity not fully visible in this matrix become apparent. First, while metagenomics and genomics appear as key descriptors, current research, such as that reviewed by Shen et al. (2026), is moving toward integrating these omics techniques with high-throughput phenotyping platforms and microfluidics to accelerate the discovery of new antimicrobials [42]. Although artificial intelligence is prominent, the focus has shifted toward specialized deep learning architectures, such as convolutional neural networks applied to microscopy images for rapid resistance detection, as well as large language models (LLMs) for mining non-indexed scientific literature, as analyzed by Chen et al. (2025) [44]. A third significant contrast is the growing importance of concepts such as the environmental exposome and urban resistome, which expand the One Health framework to include specific environmental factors and anthropogenic pressures in built environments, an approach highlighted by Yan et al. (2024) [43]. Regarding authorship, the strong Chinese representation in Figure 4 is a historical reflection, but bibliometric studies such as Alexa et al. (2025) document accelerated growth in South–South collaborations, with emerging hubs in Southeast Asia and Latin America, supported by open science agendas and scientific diplomacy [45]. Thus, the matrix in Figure 4 captures the consolidated terminological foundation, while the current frontier is defined by a tighter technical convergence (omics + microfluidics + deep AI), the quantification of the exposome, and a more diversified and collaborative geopolitics of knowledge.

The thematic map (Figure 4) reveals the conceptual structure of the field. While China and the United States dominate in publication volume, the country-level impact analysis (measured as the number of citations per article) reveals more complex patterns. For example, countries such as Denmark and the Netherlands show a disproportionately high impact, likely due to their publications in high-impact-factor journals and the fundamental nature of their methodological and critical review studies (e.g., [43]). In contrast, India’s output, although steadily increasing, exhibits a lower average citation rate. This phenomenon should not be attributed solely to a focus on local issues. Additional factors must be considered, including (1) possible language biases that limit visibility in English-indexed literature; (2) differing publication strategies, with potential preference for regional or lower-impact journals; (3) variations in citation half-life across study types; and (4) a higher proportion of local monitoring and characterization studies, which, while essential for regional management, typically achieve a shorter reach than mechanistic studies published in high-impact journals.

Evolutionary dynamics and new emphases were identified that expand and reconfigure this thematic map. First, while topics such as multi-omics and metagenomics are positioned as consolidated niches, pioneering studies such as Wu et al. (2024) integrate these platforms with spatial transcriptomics and single-cell proteomics to map resistance in complex microbiomes with unprecedented cellular and spatial resolution, creating a new niche subtopic of greater specialization [46]. Second, although artificial intelligence and machine learning appear as central topics, their recent development has branched into emerging high-density subthemes such as federated learning for privacy-preserving health data and physics-informed neural networks for modeling the environmental dispersion of contaminants, as detailed by Hou et al. (2025) [47]. These approaches do not yet appear in the map, indicating a branching beyond the analyzed period. A third crucial contrast is observed in the category of emerging topics. The current map may not fully capture the rise of concepts such as circular bioeconomy in pharmaceutical waste management or the use of digital twins to simulate the evolution of AMR in hospital environments—topics that Coronado et al. (2025) identify as drivers of translational research [48]. Finally, the categorization of climate change as a high-density niche topic coincides with recent findings [48]. However, studies such as Nneoma et al. (2025) show its fusion with planetary health and antimicrobial stewardship to form a transversal metatheme of greater centrality [49]. Thus, the strategic map provides a valid snapshot of the consolidated thematic architecture as of 2025, but frontier research is actively reshaping these categories through technological hyperspecialization; the fusion of conceptual frameworks (e.g., planetary health); and the emergence of hybrid topics at the interface of data science, systems biology, and the circular economy.

## 4. Future Research Trends

The literature applying artificial intelligence for climate action and molecular methods for combating antimicrobial resistance (AMR) is fragmented. To overcome this fragmentation and respond to the synergistic nature of these crises, future research must evolve toward more integrated, systemic, and implementation-oriented paradigms [50]. The following outlines the main emerging trends and directions.

### 4.1. Deep Technological Convergence: From Parallel Platforms to Intelligent Hybrid Systems

Cutting-edge research will no longer focus on the isolated development of AI algorithms or omics techniques but rather on their operational fusion into comprehensive diagnostic and action platforms [36,50].

#### 4.1.1. Intelligent Biosensors and Real-Time Environmental Monitoring

The development of IoT sensor networks equipped with molecular detection components (e.g., aptamers and CRISPR assays) and on-site AI processing capabilities (edge computing) will be accelerated [51]. These systems will not only detect pathogens or resistance genes in water, soil, or air but also contextualize findings with real-time climatic variables (temperature, pH, and humidity). This will transform environmental surveillance from reactive to predictive, identifying critical hotspots of AMR spread under different climate scenarios [52].

#### 4.1.2. Causal Predictive Modeling and Digital Twins

Beyond correlational models, the next frontier lies in building causal models and digital twins of ecosystems. These models will integrate metagenomic resistome data, historical and projected climate parameters, and contaminant flow data [21]. Using explainable AI (XAI) and physics-informed neural networks, they will simulate and quantify the impact of specific interventions (e.g., changes in wastewater treatment and antimicrobial use policies in agriculture) on AMR’s environmental burden and associated GHG emissions, providing a robust scientific basis for decision making [53]. Although federated learning does not appear as a high-frequency term, its necessity emerges from the Bioinformatics and Data Science cluster identified in the conceptual map, providing an ethical solution to the data fragmentation observed along the axis of international collaboration.

### 4.2. New Conceptual Frameworks: From One Health to Planetary Health and Circular Economy

Research will transcend the One Health framework toward more holistic and operational visions that recognize planetary boundaries.

#### 4.2.1. Integrating AMR into Planetary Health Frameworks

Future research must explicitly quantify how AMR dynamics affect and are affected by Earth system processes such as the carbon cycle, biodiversity loss, and alterations in biogeochemical cycles [54]. For example, studying how the dissemination of resistance genes in soils impacts fertility and carbon capture will reveal feedback loops between environmental degradation and the health crisis [39].

#### 4.2.2. Closing Cycles and the Pharmaceutical Circular Bioeconomy

Research will prioritize technologies for the valorization and advanced degradation of antimicrobials in effluents. This includes developing bioprocesses and advanced materials (e.g., catalytic nanomaterials and engineered microbial consortia) for efficient contaminant removal, as well as green chemistry strategies for designing biodegradable drugs [48,55]. The goal is to transition from a linear pharmaceutical economy to a circular one that minimizes environmental selective pressure and the sector’s carbon footprint.

### 4.3. Governance, Ethics, and Collaboration in a Globalized Science

Technical complexity must be accompanied by innovation in knowledge governance and collaboration.

#### 4.3.1. Open, Federated, and Ethical Data Science

Standards and platforms will emerge for the secure and federated exchange of sensitive data (clinical, environmental, and genomic) [56]. Federated learning will enable powerful AI models to be trained without centralizing data, thereby preserving privacy. In parallel, algorithmic ethics will become a crucial field of study, auditing and mitigating biases in predictive models that could exacerbate health or environmental inequalities across regions [19].

#### 4.3.2. Inclusive Collaboration Networks and Scientific Diplomacy

The future collaboration map (Figure 3) must be more inclusive. This requires fostering South–South consortia and supporting endogenous capacity building in regions with high climate vulnerability and AMR burden but limited representation in the literature [57]. Scientific diplomacy will be key to aligning global research agendas with local needs and integrating non-traditional actors (local communities, private technology sector, and civil society organizations) into the co-creation of solutions [58].

### 4.4. Emerging Cross-Cutting Research Lines

Based on the analysis, specific integrative research lines are proposed:

- **Development of Climate Smart Antimicrobials**: The use of AI for the in silico design of new antimicrobials and phage therapies, considering from the outset their efficacy, low propensity to generate resistance, biodegradability, and minimal carbon footprint in production [59].
- **Precision Agriculture for AMR Mitigation**: The integration of remote sensors, satellite imagery analyzed with AI, and soil molecular profiles to optimize antimicrobial use in livestock and aquaculture, reducing emissions and selective pressure [60].
- **Early Warning Systems for Epidemiological–Environmental Risk**: The development of platforms that merge seasonal climate models, antimicrobial sales data, and environmental metagenomic surveillance to predict and geolocate outbreaks of resistant infections associated with extreme climate events [61,62].

The future path requires abandoning disciplinary silos and adopting a complex systems approach [63]. Research must not only describe the interconnection between the climate and AMR but also quantify, model, and ultimately intervene through deeply integrated frontier technologies [64,65]. Success will depend on building a scientific community as interconnected and resilient as the systems that it seeks to protect.

### 4.5. Qualitative Synthesis of Key Chemical and Molecular Advances

To complement the quantitative bibliometric analysis, a qualitative meta-analysis was performed on 50 highly relevant papers (see Supplementary Table S1). The selection of these 50 papers followed a two-step process: (i) first, the 100 most cited articles within the filtered corpus were identified; (ii) second, from these, 50 articles were manually selected based on their explicit focus on chemical compounds (e.g., specific antibiotic classes and heavy metals), analytical techniques (e.g., LC-MS/MS and metagenomics), or nanomaterials. This selection ensured a representative cross-section of the chemical and molecular dimensions central to the research at the intersection of AI, climate action, and AMR.

#### 4.5.1. Chemical Compounds and Families

The analyzed studies predominantly focused on several classes of chemical compounds. In the context of AMR, beta-lactam antibiotics (penicillins and cephalosporins), fluoroquinolones (ciprofloxacin), tetracyclines, and sulfonamides were the most frequently investigated, reflecting their widespread use and environmental persistence (e.g., [45,52]). Macrolides and glycopeptides (vancomycin) also appeared, particularly in clinical resistance studies [64]. In environmental samples, co-occurrence with heavy metals (e.g., Cu, Zn, Hg, and As) was a recurring theme, as these metals can co-select for antibiotic resistance genes [31,43].

#### 4.5.2. Analytical Techniques and Molecular Methods

The methodological landscape is dominated by high-resolution mass spectrometry (HRMS) coupled with liquid or gas chromatography (LC-MS/MS, GC-MS) for quantifying antibiotics and their transformation products (e.g., [27,32]). Metagenomics and metatranscriptomics (often via next-generation sequencing) are the workhorses for profiling resistance genes and microbial communities [25,26,66]. Targeted qPCR remains common for quantifying specific genes. Emerging techniques include CRISPR-based diagnostics for rapid, point-of-care detection of resistance genes [9,67] and surface-enhanced Raman spectroscopy (SERS) for fingerprinting bacterial strains and resistance phenotypes [2,68].

#### 4.5.3. Nanomaterials and Advanced Materials

A significant portion of the frontier research involves the development of novel materials. Metal nanoparticles (Ag, Au, and ZnO) are widely explored for their antimicrobial properties and as sensing platforms [17,51]. Carbon-based nanomaterials (graphene and carbon nanotubes) are used in electrochemical biosensors [2]. Metal organic frameworks (MOFs) are gaining attention for drug delivery, catalysis, and the adsorption of antibiotics from water [41]. The synthesis and functionalization of these materials often involve green chemistry principles, aiming to reduce environmental impact.

#### 4.5.4. Integration of Artificial Intelligence with Chemical Data

AI and machine learning are being applied to chemical data in several innovative ways. Quantitative structure–activity relationship (QSAR) models, enhanced by deep learning, are used to predict the activity and toxicity of new antimicrobial compounds [19,50]. Machine learning algorithms are used to analyze complex spectral data (from MS or Raman spectra) to rapidly identify resistance patterns [2,53]. In environmental chemistry, AI is used to help predict the fate and transport of pollutants and to optimize remediation strategies [17,47]. The fusion of AI with molecular dynamics simulations is opening new avenues for understanding drug–receptor interactions at the atomic level [3].

#### 4.5.5. Research Gaps and Opportunities

Despite progress, several gaps remain. There is a need for more studies on the transformation products of antibiotics under environmental conditions and their toxicity. The interaction of chemical pollutants and resistance genes is still poorly understood. In material science, the scalability and real-world applicability of many nanosensors are yet to be demonstrated. Finally, the integration of multi-omics data with AI for a systems-level understanding of resistance evolution in complex environments is a promising but underdeveloped area.

### 4.6. Genotypic Versus Phenotypic Methods for AMR Detection: Complementary Advantages and Application Scenarios

The findings of this documentary analysis reveal a predominance of studies using molecular methods (metagenomics, qPCR, and sequencing) for the detection of antimicrobial resistance genes. However, as the recent literature rightly points out, genotypic detection has inherent limitations: the presence of a resistance gene does not guarantee its phenotypic expression due to regulatory mechanisms, gene silencing, or environmental conditions that modulate expression [50]. Conversely, phenotypic resistance can occur in the absence of known resistance genes, mediated by mechanisms such as persister tolerance, biofilm formation, or epigenetic modifications [63]. In response to these limitations, rapid phenotypic methods for AMR detection have gained significant ground in recent years. These methods are based on the direct measurement of bacterial responses to antibiotics, providing a functional view of resistance. Among the most promising technologies are the following:

- **Metabolic activity-based assays**: These detect changes in bacterial metabolism (e.g., ATP production and redox activity) in the presence of antibiotics, enabling susceptibility determination within hours. A notable example is the development of microfluidic platforms that monitor bacterial growth at the single-cell level [59].
- **Enzymatic assays**: These detect the activity of resistance-encoding enzymes, such as β-lactamases, using chromogenic or fluorogenic substrates, offering rapid and specific results [60,61].
- **DNA quantification following antibiotic exposure**: Methods such as the one described in [64] quantify bacterial DNA via digital PCR after a brief incubation period with antibiotics, enabling phenotypic susceptibility determination in under 30 min.
- **Microfluidic platforms and biosensors**: Microfluidics has emerged as a key technology for integrating multiple functions (culture, antibiotic exposure, and detection) into compact devices, enabling high-throughput rapid phenotypic assays with minimal sample volumes [65,66]. These systems can be coupled with optical, electrochemical, or spectrometric detection to quantify bacterial response in real time.

The clear conceptual distinction that we have outlined in this work between genotypic detection (the presence of ARGs via PCR or metagenomics) and phenotypic expression (functional resistance assessed through culture and antibiograms) is equally applicable and critical in veterinary and agricultural contexts. Implementing integrated One Health surveillance systems will require harmonizing these approaches across all compartments, including animal, human, and environmental compartments. For example, the early detection of a high-concern ARG (such as *mcr-1* or *bla*NDM) in farm samples using rapid genotypic methods could provide an alert of an emerging problem before it manifests clinically. However, such genotypic alerts must be accompanied by phenotypic studies to understand the current magnitude of functional resistance and its impact on animal health and treatment efficacy. The development of biosensors and low-cost technologies, such as those discussed in the context of treatment plants, has enormous potential to be adapted and deployed in low- and middle-income countries (LMICs), where AMR surveillance in farms is both most needed and most challenging.

**Comparative advantages and complementarity**: Genotypic and phenotypic methods should not be viewed as mutually exclusive but rather as complementary. The following table summarizes their advantages and optimal application scenarios (Table 5).

Implications for frontier technology integration: The convergence of rapid phenotypic methods with artificial intelligence and microfluidic platforms represents one of the most promising frontiers in the fight against AMR. Microfluidic systems generate large volumes of data (images and real-time signals) that can be analyzed using machine learning algorithms to identify growth patterns, detect resistance events at the single-cell level, and optimize assay protocols [38]. Furthermore, combining phenotypic and genotypic data into integrated models could enable a more comprehensive understanding of resistance and improve diagnostic accuracy. In the context of climate change, rapid phenotypic methods also have relevant applications: they can be used to monitor the impact of environmental factors (temperature, pH, and pollutants) on resistance expression in environmental microbiomes, helping to elucidate how climate change modulates the AMR threat [67]. Future research should explore the integration of microfluidic platforms with nanomaterial-based biosensors and AI algorithms to create environmental surveillance and clinical diagnostic systems that combine genotypic specificity with the functional relevance of phenotypic methods.

## 5. Materials and Methods

This documentary analysis was grounded in a systematic methodology designed to identify, select, and examine scientific literature converging on the application of artificial intelligence (AI) for environmental sustainability and molecular methods to address antimicrobial resistance (AMR), with a focus on chemical and molecular aspects.

### 5.1. Data Sources and Search Strategy

Scopus was selected as the primary data source because it is the largest abstract and citation database; it was also chosen because of its broad multidisciplinary coverage and the quality of its indexed metadata, which are essential for a frontier topic spanning chemistry, environmental sciences, computational sciences, and biomedicine. The search was conducted in the Scopus database on 15 January 2026, using the following search equation: (TITLE-ABS-KEY (artificial intelligence” OR “ai” OR “machine learning” OR “deep learning”) AND (“environmental management” OR “environmental monitoring” OR “sustainability” OR “ecosystem”) AND (“antibiotic resistance” OR “antimicrobial resistance” OR “resistance detection” OR “pathogen resistance”) AND PUBYEAR > 1999 AND PUBYEAR < 2026 AND (LIMIT-TO (LANGUAGE, “English”)). This equation combines three core concepts: (1) artificial intelligence and machine learning technologies, (2) environmental management and climate sustainability, and (3) antimicrobial resistance and molecular detection methods. For clarity, the term “environmental management” refers to governance frameworks and strategies for overseeing human impacts on ecosystems; “environmental sustainability” denotes the long-term goal of maintaining ecological balance and resource availability; and “environmental chemistry” is the technical approach focused on the analysis of chemical pollutants (e.g., antibiotics and heavy metals) and their transformation products in environmental matrices.

**Figure 5:**
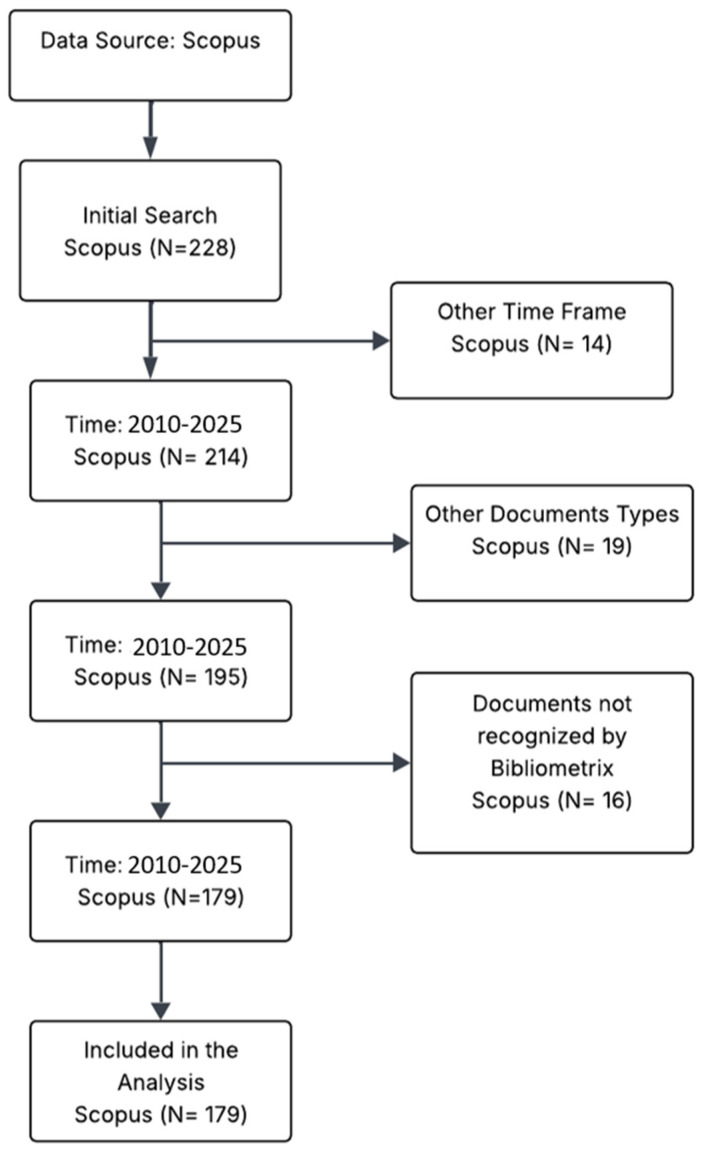
Process of filtering and selecting documents from Scopus for analysis (2010–2025).

As previously mentioned, the search was conducted in the Scopus database, which was selected for its broad coverage of the chemical, environmental, and biomedical sciences, as well as for the quality of its metadata for bibliometric analyses. The search period spanned from 2010 to 2025. The search strategy combined terms related to artificial intelligence, environmental chemistry, and antimicrobial resistance, restricting results to articles and reviews published in English. The selection process followed the PRISMA 2020 guidelines (Figure 5). The initial search retrieved 228 records. After applying automatic filters for publication year (2010–2025) and document type (articles and reviews), 14 records were removed, leaving 214 records for the screening phase. In this phase, titles and abstracts were reviewed, and 15 records were excluded because they did not address the intersection of the three topics (AI, environmental chemistry, and AMR), resulting in 199 records eligible for full-text assessment. During the full-text evaluation, 20 documents were excluded: 10 due to metadata issues (not processable by the Bibliometrix tool, v5.3.0) and 10 for lacking a sufficiently detailed chemical or molecular focus. This criterion was assessed by verifying the explicit presence of at least one of the following in the title, abstract, or methodology: (a) specific antibiotic classes (e.g., beta-lactams, fluoroquinolones, and tetracyclines); (b) heavy metals (e.g., Cu, Zn, and As); or (c) analytical techniques (e.g., LC-MS/MS, qPCR, and metagenomics). Studies exclusively clinical or epidemiological without any such chemical or molecular data were excluded.

### 5.2. Data Extraction and Analysis

For quantitative analysis, bibliographic data (authors, titles, journals, citations, affiliations, and keywords) were exported from Scopus and processed using R Studio (bibliometrix package) and VOSviewer for network visualization. Impact metrics such as the total number of citations, h-index, and average number of citations per paper were calculated. For a qualitative meta-analysis, the selected papers were read in full, and the following information was extracted into a standardized form: (i) the chemical compounds studied (e.g., antibiotics and metals); (ii) the analytical techniques used (e.g., LC-MS/MS, qPCR, and CRISPR-based assays); (iii) the type of AI application; (iv) nanomaterials or advanced materials developed; and (v) key findings related to chemical mechanisms.

### 5.3. Selection of Documents for Impact Analysis

For an analysis of the most influential documents (Table 3), the 10 articles with the highest number of total citations within the filtered corpus (n = 179) were selected. Although this metric is sensitive, for the construction of a strategic map (also known as a thematic map), which relates centrality (the relevance of a topic to the field) and density (the internal development of the topic), we followed the methodology of Cobo et al. (2014) [68]. Keyword clusters were generated from a co-occurrence network. Centrality was calculated as the total link strength of a cluster with other clusters, indicating its importance in structuring the field. Density was calculated as the average strength of the internal links within the cluster, reflecting its cohesion and maturity. Thresholds for classifying topics into quadrants were defined using the mean values of centrality and density across all clusters. Accordingly, topics in the upper-right quadrant (high centrality and high density) were classified as motor themes (well developed and central). Those in the lower-right quadrant (high centrality and low density) were basic and transversal themes. Topics in the upper-left quadrant (low centrality and high density) were highly specialized or niche themes. Finally, those in the lower-left quadrant (low centrality and low density) were emerging or declining themes.

Figure 1 was generated using VOSviewer (version 1.6.20). The network was constructed based on co-authorship relationships among authors appearing in the filtered corpus of 179 documents. The minimum number of documents per author was set to 2, resulting in 87 authors meeting the threshold. The normalization method used was “association strength,” which calculates the similarity between authors based on the number of co-authored documents relative to the expected number under random co-occurrence. The clustering algorithm employed was the VOS mapping technique with a resolution parameter of 1.0, which groups authors into clusters based on their co-authorship patterns. Node size reflects the total link strength (i.e., the sum of the association strengths of co-authorship links) of each author. Link thickness is proportional to the number of co-authored documents between two authors. Colors represent distinct clusters identified by the algorithm.

### 5.4. Eligibility Criteria and Quality Filtering

For the qualitative synthesis of chemical and molecular mechanisms (Section 4.5), a stricter quality filter was applied, selecting only studies that met at least three of the following criteria for methodological robustness and analytical depth:

- Explicit chemical characterization: The use of analytical techniques (e.g., LC-MS/MS and GC-MS) for the identification and quantification of specific antimicrobial compounds or their transformation products in environmental matrices.
- Application of advanced molecular methods: The use of metagenomics, metatranscriptomics, or targeted qPCR for the profiling of antibiotic resistance genes (ARGs) and microbial communities.
- Validation of AI methodologies: A clear description of the AI/ML algorithms used, including validation strategies (e.g., cross-validation and independent test sets) for tasks such as resistance prediction or data integration.
- Integration of AI with chemical or molecular data: Studies that demonstrate a concrete fusion of AI techniques with chemical analysis (e.g., QSAR models and spectral analysis) or molecular datasets (e.g., multi-omics integration).
- Reproducibility and data availability: The inclusion of open-source code, data repositories, or detailed experimental protocols that enable replication of the computational or analytical workflow.

Studies that did not meet these criteria were considered lower-quality evidence for the mechanistic synthesis and were used only to contextualize general trends (e.g., in country-level productivity analysis) and not as the basis for the core mechanistic conclusions. Figure 2 was generated using VOSviewer based on co-authorship relationships aggregated at the country level. The analysis included all 179 documents. The minimum number of documents per country was set to 5, and the minimum number of citations per country was set to 0 (no citation threshold was applied) to ensure the inclusion of countries with high collaborative activity but potentially lower citation counts. The normalization method was “association strength.” Node size is proportional to the number of publications originating from each country. Link thickness represents the total link strength (i.e., the intensity of co-authorship collaborations) between countries. The network layout was generated using the VOS mapping algorithm with default parameters (attraction = 2, repulsion = 1). Color assignment was performed automatically by VOSviewer based on cluster membership.

### 5.5. Construction of the Importance Matrix of Concepts (Figure 3)

Figure 3 presents an importance matrix that integrates three complementary layers of analysis: (i) terms appearing in abstracts and titles (AB_TM), (ii) author-provided keywords (DE), and (iii) author names (AU). The matrix was generated using the bibliometrix package (version 4.1) in R Studio (version 2024.12.0). For AB_TM and DE, the minimum frequency threshold was set to 10 occurrences, and the analysis was restricted to the 50 most frequent terms. Term extraction was performed using the termExtraction function with default parameters (ngrams = 1, stemming = FALSE). For author names (AU), the minimum frequency threshold was set to 3 documents per author. The importance score for each term was derived from the product of its frequency (relative occurrence) and its centrality within the co-occurrence network. The figure was visualized using the ggplot2 package, with point size scaled according to the importance score. Colors indicate the source of the term (AB_TM in blue, DE in red, and AU in green).

### 5.6. Construction of the Strategic Map of Topics (Figure 4)

Figure 4 presents a strategic (thematic) map, an analysis of which was performed using the bibliometrix package in R Studio. First, a co-occurrence network of author keywords was generated from the 179 documents. Keywords appearing in fewer than 5 documents were excluded, resulting in 142 keywords. The co-occurrence matrix was normalized using the association strength measure. Clusters of keywords were identified using the Louvain clustering algorithm (resolution parameter = 1.2). For each cluster, two metrics were calculated:

- Centrality (relevance): The sum of the normalized co-occurrence links between keywords in a given cluster and keywords in all other clusters. This metric indicates the extent to which a topic connects to other research areas.
- Density (development): The average strength of the co-occurrence links within a cluster. This metric reflects the internal cohesion and maturity of a research topic.

The topics were classified into four quadrants using the mean values of centrality and density across all clusters as thresholds: Upper right (high centrality and high density): Motor themes (well developed and central).

- Lower right (high centrality and low density): Basic and transversal themes.
- Upper left (low centrality and high density): Niche themes (highly specialized).
- Lower left (low centrality and low density): Emerging or declining themes.

The visualization was created using ggplot2, with point size representing the number of keywords per cluster and the point position determined by centrality (*x*-axis) and density (*y*-axis).

## 6. Conclusions

This documentary analysis successfully achieved its objectives by critically examining the integration of artificial intelligence for climate action and molecular methods for addressing antimicrobial resistance (AMR). It was found that publications on AI and sustainability exhibit greater maturity and visibility, with a consolidated citation volume, while AMR research, although more recent, is rapidly evolving toward the use of specialized and high-precision molecular approaches. In addition, the mapping of collaboration networks revealed a highly interconnected and transnational scientific structure, with hubs in China, the United States, Spain, and Australia, alongside the growing participation of emerging actors in regions such as India and South Korea. Likewise, the comparison of impact metrics highlights Anglo-Saxon and European leadership in the number of citations per article, while countries with high productivity, such as India, show a lower impact, pointing to differences in reach and international visibility.

The visualization of thematic patterns confirms the consolidation of research cores such as metagenomics, machine learning, and environmental surveillance while also underscoring the emergence of integrative frameworks such as One Health and planetary health. Emerging research lines that merge both fields were also proposed, emphasizing the need to address these crises in a synergistic and systemic manner. Taken together, this study provides solid bibliometric evidence validating the urgency of building interdisciplinary bridges between climate-focused AI and molecular tools, offering a foundation to guide policies, funding, and research strategies toward integrated solutions that strengthen global resilience against interconnected environmental and health challenges.

Future work should deepen operational technological convergence, developing hybrid systems that combine intelligent biosensors, ecosystem digital twins, and federated data platforms for predictive and contextualized environmental monitoring. The development of intelligent biosensors could transform environmental monitoring from reactive to predictive, potentially identifying critical points of antimicrobial resistance (AMR) spread under different climate scenarios. However, this transformation will require significant advances in miniaturization, robustness, and validation under real-world conditions. It is essential to foster more inclusive and ethical collaboration networks that incorporate vulnerable regions and non-traditional actors, ensuring that knowledge governance and scientific diplomacy accompany technical innovation for an effective and equitable global response.

## Figures and Tables

**Table 1:** Scientific articles on artificial intelligence applied to climate action: documentary analysis.

	Title	Year	Cited By	Source Title	Authors	Document Type	Abstract
1	Food Security, Safety, and Sustainability—Getting the Trade-Offs Right [24].	2020	275	Frontiers in Sustainable Food Systems	Vågholm, L. et al.	Review	The United Nations’ Sustainable Development Goals include eradicating hunger and ensuring food security for an estimated 10 billion people by 2050
2	Gammaproteobacteria, a core taxon in the guts of soil fauna, are potential responders to environmental concentrations of soil pollutants [25].	2021	95	Microbiome	Zhang, Q. et al.	Article	Ubiquitous members of the gut microbiota are acquired from the environment and contribute to host health, and the gut microbiota of soil invertebrates is gradually assembled
3	NanoARG: A web service for detecting and contextualizing antimicrobial resistance genes from nanopore-derived metagenomes [26].	2019	92	Microbiome	Arango-Argoty, G.A.; Dai, D.	Article	Direct and indirect selection pressures imposed by antibiotics as selective agents and horizontal gene transfer are fundamental drivers of evolution
4	Source identification of antibiotic resistance genes in a peri-urban river using novel crAssphage marker genes and metagenomic signatures [27].	2019	88	Water Research	Chen, H.; Bai, X.; Li, Y.; Jin, M.	Article	Antimicrobial resistance is a growing public health concern, and the environment is regarded as an important reservoir for the dissemination of antibiotic resistance genes
5	Data Analytics for Environmental Science and Engineering Research [28].	2021	84	Environmental Science and Technology	Gupta, S.; Aga, D.; Pruden, A.	Article	The advent of new data acquisition handling techniques has opened the door to alternative comprehensive approaches to environmental monitoring, thus improving our understanding
6	Advancing sustainable development goals through immunization: a literature review [29].	2021	84	Globalization and Health	Decouttere, C.; De Boeck, K.	Article	Immunization directly impacts health (SDG3) and contributes to achieving Sustainable Development Goals (SDGs) 14 and 17
7	Statistical and machine learning techniques in human microbiome studies: contemporary challenges and solutions [30].	2021	82	Frontiers in Microbiology	Moreno-Indias, I.; Lahti, L.	Review	The human microbiome has emerged as a central research topic in human biology and biomedicine, and current microbiome studies have generated high-throughput data
8	Organic fertilization co-selects genetically linked antibiotic and metal(loid) resistance genes in global soil microbiome [31].	2024	78	Nature Communications	Liu, Z.-T.; Ma, A.; Zhu, Y.-G.	Article	Antibiotic resistance genes (ARGs) and metal(loid) resistance genes (MRGs) coexist in organic fertilizer-based agroecosystems
9	Hospital discharges in urban sanitation systems: Long-term monitoring of wastewater resistome and microbiota in relationship to their eco-exposome [32].	2020	74	Water Research X	Buelow, E.; Rico, A.; Gasco, L.	Article	Wastewaters are important sources of the dissemination of antimicrobial resistance (AMR) genes in the environment, and hospital wastewater contains high loads of micro-pollutants
10	Applications of Smart Technology as a Sustainable Strategy in Modern Swine Farming [33].	2022	72	Sustainability (Switzerland)	Mahfuz, S.; Mun, H.-S.; D’Souza, D.	Article	The size of the pork market is increasing globally to meet the demand for animal protein, resulting in larger-sized swine farms

**Table 2:** Main authors applying AI in climate studies and molecular methods in AMR studies, according to citation and collaboration metrics (2010–2025).

Author	ID Scopus	Primary Affiliation	Total Citations	Citations of Most Cited Document	Average Citations per Article	Number of Contributors
Zhu, Yongguan	7406073704	Institute of Urban Environment, Chinese Academy of Sciences, Beijing, China	240	95	60	28
Zhang, Qi	55699339400	ARC Centre of Excellence in Synthetic Biology, Macquarie University, Sydney, Australia	185	95	46.25	29
Zhang, Zhenyan	57194409451	Centre de Recerca Ecològica i Aplicacions Forestals (CREAF-CERCA), Barcelona, Spain	172	95	57.33	19
Qian, Haifeng	35235046800	Unknown	172	95	57.33	19
Peñuelas, Josep J.	7006747772	Research Center for Eco-Environmental Sciences, Chinese Academy of Sciences, Beijing	162	95	54	24
Pruden, Amy J.	57049332700	Virginia Tech College of Engineering, Blacksburg, VA, United States	176	92	88	7
Zhang, Liqing	55709248500	Virginia Tech College of Engineering, Blacksburg, VA, United States	176	92	88	7
Vikesland, Peter J.	6603770862	Virginia Tech College of Engineering, Blacksburg, VA, United States	176	92	88	7
Lu, Tao	57224808283	Universitat Autònoma de Barcelona, Barcelona, Spain	118	95	59	12
Zhu, Dong	57190734423	Universitat Autònoma de Barcelona, Barcelona, Spain	91	78	45.5	16

**Table 3:** Scientific output, citations, and impact by country in frontier technologies for climate and AMR.

	Country	Papers	Total Citations	Average Citations	Affiliations	Funded Papers	Funders	Impact (Citations/Paper)
1	United States	67	1116	16.66	67	45	14	16.66
2	China	48	651	13.56	48	39	5	13.56
3	India	33	180	5.45	33	15	5	5.45
4	United Kingdom	26	701	26.96	26	18	11	26.96
5	Italy	12	157	13.08	12	4	1	13.08
6	Spain	10	328	32.8	10	8	5	32.8
7	Australia	10	128	12.8	10	7	4	12.8
8	Saudi Arabia	8	93	11.62	8	6	1	11.62
9	Germany	8	172	21.5	8	6	5	21.5
10	Canada	6	120	20	6	5	6	20

**Table 4:** Scientific output and impact of leading journals in AMR, environmental microbiology, and sustainability (2010–2025).

Source	Articles	H-Index	G-Index	M-Index	TC	NP	PY Start	Quartile (JCR 2024)
Science of the Total Environment	12	9	12	1.5	450	12	2015	Q1
Frontiers in Microbiology	10	7	10	1.0	320	10	2016	Q1
Antibiotics	9	6	9	1.2	210	9	2018	Q2
Environmental Science & Technology	8	7	8	1.0	580	8	2014	Q1
Water Research	7	6	7	0.9	390	7	2013	Q1
Nature Communications	5	5	5	0.8	620	5	2017	Q1
ACS Synthetic Biology	4	4	4	0.8	185	4	2019	Q1
Microbiome	4	4	4	0.6	210	4	2016	Q1
Journal of Hazardous Materials	4	3	4	0.5	98	4	2018	Q1
Chemosphere	3	3	3	0.4	76	3	2015	Q2

**Table 5:** Comparison of genotypic and rapid phenotypic methods for AMR detection.

Aspect	Genotypic Methods	Rapid Phenotypic Methods
What they detect	Genetic potential for resistance	Functional expressed resistance
Main advantage	Identification of resistance mechanisms, molecular epidemiology, surveillance of emerging genes	Direct susceptibility determination, immediate clinical relevance
Limitation	Does not inform about expression; may yield false positives/negatives	May require prior culture; less mechanistic information
Optimal scenario	Epidemiological surveillance, environmental studies, detection of novel genes	Rapid clinical diagnosis, treatment guidance, point-of-care susceptibility testing
Integration with AI	Resistance prediction from genomic data (QSAR models, machine learning)	Image analysis, sensor signal processing, assay condition optimization

## Data Availability

The original contributions presented in this study are included in the article. The bibliographic data supporting the findings were retrieved from the Scopus database. The processed datasets (CSV format), the R scripts used for the bibliometric analysis, and the VOSviewer network files are available from the corresponding author upon reasonable request.

## Supplementary Materials

The following supporting information can be downloaded at: https://www.mdpi.com/article/10.3390/molecules31101571/s1.
