# Supplementary material for: Mapping the Convergence of Frontier Technologies for Major Environmental Challenges: A Chemical and Molecular Perspective on the Use of AI for Climate Action and Antimicrobial Resistance
Source: Molecules. 2026 May 8;31(10):1571. doi: 10.3390/molecules31101571 (PMC13210365; doi:10.3390/molecules31101571)
Supplement: Supplementary file 1 [file molecules-31-01571-s001.zip › molecules-4188427-supplementary.pdf]

Table S1. Qualitative analysis of 58 selected studies: chemical compounds, analytical techniques (genotypic and phenotypic), microfluidic platforms and artificial intelligence applications at the climate change-antimicrobial resistance interface.

	No. Ref	Reference (Autor, Año, Revista)	Chemical Compounds	Analytical Techniques	AI Application	Nanomaterials	Key Findings
**1**	52	Chambial P, 2025, ARCH MICROBIOL	Big data, AI, cloud computing	Conceptual Framework	Agentic AI, Large Language Models, Explainable AI (XAI)	Not specified	Proposes an updated framework (Ø4VsÆ) for big data in healthcare, emphasizing veracity (explainability), validity (fairness), and viability (sustainability) in the age of AI.
**2**	8	Patra M, 2025, REV ENVIRON CONTAM TOXICOL	Bisphenol A (BPA), ESR1, PTGS2, CCL2, FLNA, TRPV1	Network Toxicology, Molecular Docking (AutoDock), qRT-PCR	CANDO platform for de novo drug prediction	Not specified	Identified BPA's molecular mechanisms in osteoarthritis (OA) via hub targets (ESR1, PTGS2, etc.) and predicted 14 potential OA treatments (e.g., glucosamine, ibuprofen) using computational drug discovery.
**3**	15	Ahlawat U, 2024, J EXP AGRIC INT	Not specified	Multi-omics (genomics, transcriptomics, proteomics, metabolomics, phenomics), CRISPR/Cas, GWAS, Genomic Selection (GS)	AI, Machine Learning (ML) for trait forecasting and data-driven insights	Not specified	Highlights the integration of multi-omics, gene editing (CRISPR/Cas), and AI/ML for developing climate-resilient, high-yielding crops. Emphasizes a systems biology approach combining speed breeding.
**4**	2	Taha BA, 2024, ACS SYNTH BIOL	Lead (Pb), Zinc	Cell-free gene expression, Fluorescence assays, Inductively coupled plasma mass spectrometry	Active learning-guided optimization (ML)	Nanoparticles (implied in biosensor context)	Developed a multi-objective, ML-guided workflow to engineer a cell-free biosensor (PbrR) for detecting lead in water at ~5.7 ppb, enhancing sensitivity and selectivity away from zinc.

**5**	56	Vashishth TK, 2025, AI-POWERED ADV PHARMACOL	Vanillic acid 4-β-D-glucoside, schaftoside, rutin, phenolic compounds, flavonoids	Metabolomics (UHPLC-QTOF-MS, RP-HPLC, UPLC-QQQ-MS), in-vitro bioassays	Machine Learning (Random Forest Regression)	Not specified	ML-driven metabolomics identified July as optimal harvest time for *D. officinale* leaves and key bioactive compounds (vanillic acid glucoside, schaftoside, rutin) responsible for anti-inflammatory/antioxidant effects.
**6**	57	Jain R, 2025, SSRN	Not specified	Genomics, Pangenomics, Metagenomics, Transcriptomics	AI-based methods	Not specified	Reviews how omics tools (genomics, pangenomics, transcriptomics) can enhance quantitative microbial risk assessment (QMRA) by accounting for genetic heterogeneity of foodborne pathogens, improving food safety decisions.
**7**	24	Vågsholm I, 2020, FRONT SUSTAIN FOOD SYST	Not specified	Not specified	Machine Learning, Computer Vision, Blockchain, AI	Not specified	Reviews the synergy of microbial biotechnologies (precision fermentation) with AI tools (ML, computer vision) across the food supply chain (Food System 5.0), focusing on energy sustainability and food waste valorization.
**8**	39	Karnwal A, 2025, FRONT MICROBIOL	Not specified	Smart indicators (TTI, pH, humidity), biosensors, sensor systems	Artificial Intelligence (AI), Internet of Things (IoT)	Nanotechnology	Reviews the integration of smart indicators and sensor technologies with IoT, AI, and nanotechnology for real-time detection of food spoilage and contamination, promoting transparency and sustainability in food safety.
**9**	4	Naga NG, 2025, TROP DIS TRAVEL MED VACCINES	Not specified	Portable biosensors, wearable sensors	Artificial Intelligence (AI)	Not specified	Explores how accessible analytical tools and AI are transforming analytical chemistry into a more inclusive discipline (citizen science), enabling public participation in environmental monitoring and food quality assessment.

**10**	40	Oladipo EK, 2025, MICROB PATHOG	Not specified	Photoelectric sensors, multimodal sensing	Artificial Intelligence (AI)	Photoelectric nanozymes (PENz) (noble metals, metal oxides, carbon-based, MOFs)	Reviews the evolution of food safety sensors from monomodal to multimodal using photoelectric nanozymes, enhanced by AI for data analysis and pollutant prediction, creating intelligent monitoring systems.
**11**	31	Liu ZT, 2024, NAT COMMUN	Emerging contaminants (hydrocarbons, pesticides, pharmaceuticals)	Genomics, Transcriptomics, Proteomics, Metabolomics	Artificial Intelligence (AI), Machine Learning (ML)	Sensors (implied)	Discusses integrating multi-omics with sensors, digital twins, and AI for adaptive and precision bioremediation of soil contaminants, enabling real-time monitoring and targeted interventions.
**12**	25	Zhang Q, 2021, MICROBIOME	Antibiotics (multi-antibiotic wastewater), Nitrous oxide	Metagenomics, Machine Learning (ML), Structural Equation Modeling	Machine Learning (Random Forest)	Biochar, pyrite	Reveals a trade-off in constructed wetlands: biochar removes pollutants well but creates resistance gene hotspots, while pyrite balances high performance with low ecological risk by suppressing antibiotic resistance genes.
**13**	1	Lawal OP, 2025, CUREUS	Drug compounds	Computational analysis	Deep Learning (Parallel CNN-BiLSTM with attention)	Not specified	Proposes EDTA, a deep learning model combining parallel CNNs and BiLSTM for efficient and accurate drug-target affinity prediction, outperforming state-of-the-art methods with fewer parameters.
**14**	27	Chen H, 2019, WATER RES	Antibiotics (e.g., sulfaphenazole), PAHs (e.g., acenaphthene), PCBs	H2O AutoML, Metagenomics	Causal Machine Learning (Double Machine Learning), Interpretable ML	Not specified	Used causal ML to identify synergistic effects among pollutants driving antibiotic resistance genes (ARGs) in drinking water, finding that antibiotics and PAHs increase ARGs while PCBs suppress them.
**15**	42	Shen M, 2026, SENS DIAGN	Sulfamethoxazole (SMX), ammonia nitrogen	Machine Learning (Random Forest Regression), Microcosm experiments	Machine Learning (Random Forest, feature importance, causal analysis)	Not specified	ML framework (Random Forest) identified key process control parameters (acclimation period, pH) governing sulfamethoxazole biodegradation in activated sludge, validated by microcosm experiments.

**16**	13	Islam SI, 2024, J WORLD AQUAC SOC	Antibiotics (e.g., tetracycline)	Adsorption, Photocatalysis, Ozonation, Fenton oxidation, Electrochemical oxidation	AI-optimized processes	Nanomaterials (targeted adsorbents, photocatalysts), Ozone nano-bubbles	Reviews physicochemical strategies for antibiotic removal in aquaculture wastewater, highlighting synergistic processes (e.g., nano-bubbles with photocatalysis) and the future role of AI and lifecycle assessments.
**17**	33	Mahfuz S, 2022, SUSTAINABILITY	Not specified	Solar desalination systems	Artificial Intelligence (AI) for system optimization, predictive modeling	Nanomaterials, Nano-fluids	Comprehensive review of solar desalination advancements, including system innovations (reflectors, wicks, collectors) and nanomaterial integration, with a focus on AI for optimizing performance and achieving sustainability goals.
**18**	26	Arango-Argoty GA, 2019, MICROBIOME	Sulfonamide antibiotics	Adsorption experiments	Machine Learning (Gradient Boosting, ANN, Gaussian Process Regression), Explainable AI (SHAP)	Biochar	Applied explainable ML to predict sulfonamide adsorption on biochar, identifying BET surface area as the dominant driver, thus providing guidance for biochar evaluation in water treatment.
**19**	50	Behera B, 2025, ARCH MICROBIOL	Not specified	Soil enzyme assays, Metagenomics, Transcriptomics	Artificial Intelligence (AI)-driven analytics	Biochar	Reviews the role of soil enzymes as bioindicators of soil health, emphasizing the potential of AI and enzyme engineering for optimizing nutrient cycling and pollutant degradation in sustainable agriculture.
**20**	55	Ortiz-Gómez V, 2025, ACS OMEGA	Phenolic compounds (PCs), Bisphenol A	Biosensing (colorimetric, fluorometric, electrochemical)	Deep Learning, AI for intelligent systems	Nanozymes (various)	Systematically reviews nanozyme-based biosensors for detecting phenolic contaminants, covering catalytic mechanisms, sensor design, and future intelligent monitoring systems incorporating AI.

**21**	54	Singh S, 2025, ECOHEALTH	Not specified	Genomics, Transcriptomics, Proteomics, CRISPR/Cas9, High-throughput sequencing	Machine Learning, Next-gen tools for precision breeding	Not specified	Discusses using multi-omics and ML to enhance biotic stress tolerance in underutilized small millets, identifying pathways, genes, and markers for developing disease-resistant varieties.
**22**	48	Coronado-Contreras SA, 2025, BIOMASS	Polycyclic aromatic hydrocarbons (PAHs), Heavy metals (HMs)	Microbial kinetics, metagenomics, CRISPR-Cas gene editing	Artificial Intelligence (AI)-driven monitoring systems	Not specified	Reviews microbial bioremediation strategies for PAH-heavy metal co-contamination, highlighting the use of gene editing (CRISPR-Cas), AI for monitoring, and algae-microbe consortia for enhanced remediation.
**23**	49	Nneoma UC, 2025, FRONT ENERGY RES	Antibiotics	Molecular Dynamics (MD) simulations, Density Functional Theory (DFT)	Machine Learning (ML)-accelerated MD	Microplastics	Critically reviews antibiotic-microplastic interactions using MD and DFT, proposing a framework for standardizing simulations and integrating ML to predict environmental risks and antibiotic resistance.
**24**	53	Abidharini JD, 2025, CRC PRESS	Not specified	Genomics, Proteomics, Transcriptomics, Metabolomics, Phenomics, Epigenomics	Artificial Intelligence (AI), Machine Learning (ML)	Not specified	Reviews the use of multi-omics and AI/ML for deciphering plant stress responses, emphasizing data integration, standardization, and the potential for developing stress-competent crops.
**25**	3	Sharma P, 2025, ACADEMIC PRESS	Amino acids, sugars, phospholipids	Cell-growth-coupled, optical and electrochemical biosensors	AI-optimized, intelligent biosensors	Not specified	Reviews engineered biosensors in food biomanufacturing for monitoring nutrient synthesis (e.g., amino acids). Highlights future integration with AI for multiplexing, smart sensors, and cross-species platforms.
**26**	51	Angeles Flores G, 2025, MICROORGANISMS	Not specified	Not specified	AI-guided rational molecular design	Antimicrobial polymers (peptide-mimetic,	Reviews recent advances in designing antimicrobial polymers with diverse uptake/killing mechanisms, emphasizing AI-guided design, high-

						glycosylated, non-charged)	throughput screening, and translationally relevant principles to combat antimicrobial resistance.
**27**	20	Susilawati A, 2025, INDONES J SCI TECHNOL	Not specified	Bibliometrics (Biblioshiny, VOSviewer, QDA Miner)	Artificial Intelligence (AI), Machine Learning (ML)	Sensors, biosensors	Explores the convergence of digital technologies (AI, ML, IoT) with bioeconomy concepts (biomass, biotechnology) to create a framework for comprehensive measurement and assessment of the bioeconomy.
**28**	58	Syed MA, 2025, CRC PRESS	PAHs, heavy metals	Spectroscopic methods (μ-FTIR, Raman), thermal analysis (Py-GC/MS)	AI-assisted analysis	Not specified	Comprehensive review on microplastic migration, biotoxicity, and biodegradation in terrestrial ecosystems. Proposes multi-technique integration and AI-assisted analysis as future directions for detection.
**29**	7	Xavier SP, 2025, DISCOV PUBLIC HEALTH	Heavy metals (MeHg), PCBs, phthalates, microbial toxins	Metabolomics, FTIR, Raman spectrometry, LC-MS, high-throughput sequencing	AI-based exposure diagnostics	Microplastics	Synthesizes knowledge on marine contaminants (microplastics, heavy metals, POPs) and their transfer through food webs to humans, highlighting the role of AI-based diagnostics and multi-omics in future risk assessment.
**30**	6	Livingston G, 2024, LANCET	Not specified	Not specified	Artificial Intelligence (AI)	Not specified	Discusses the global epidemiology of sepsis, including the role of AI, climate change, and antimicrobial resistance, while highlighting challenges in low- and middle-income countries.
**31**	38	Mee RWM, 2025, CONTEMP EDUC TECHNOL	Phytopathogens	Electrical, electrochemical, chemiresistive, optical biosensors	Artificial Intelligence (AI), Machine Learning (ML), Network Analysis	Functional nanomaterials (various)	Reviews functional nanomaterial-based biosensors for plant disease detection, emphasizing their integration with AI, ML, and IoT as adaptive nodes in complex agricultural systems for sustainable farming.

**32**	41	Guo J, 2025, J NANOBIOTECHNOL	Microplastics, PFAS, pharmaceuticals, engineered nanomaterials (ENMs)	CRISPR-based biosensors, high-resolution spectroscopy	Machine Learning (ML) for contamination mapping	Biochar, nanomaterials	Reviews the impact of emerging contaminants on agroecosystems, highlighting advanced detection tools (CRISPR biosensors, ML mapping) and sustainable remediation strategies (phytoremediation, biochar, nano-enabled degradation).
**33**	47	Hou Q, 2025, J HYDROL	Heavy metals, pathogens, micropollutants	Localized surface plasmon resonance (LSPR), SERS, photocatalysis	AI-assisted design and optimization	Plasmonic nanomaterials	Examines plasmonic nanomaterials for water purification, desalination, and monitoring via LSPR and SERS. Discusses integration with IoT and AI for smart water quality networks and future challenges.
**34**	44	Yan W, 2024, ENVIRON INT	Not specified	DNA barcoding (ITS), Phylogenomics, Multi-omics (genomics, transcriptomics, proteomics, metabolomics)	AI-driven data analytics	Not specified	Reviews progress in fungal systematics using DNA barcoding and phylogenomics, highlighting the potential of AI-driven analytics and multi-omics for understanding fungal diversity and adaptation.
**35**	28	Gupta S, 2021, ENVIRON SCI TECHNOL	Heavy metals, organic pollutants	Plant-derived probes (flavonoids, phytochelatins), soil enzyme inhibition assays	AI-integrated arrays	Paper-based strips, microfluidic devices (implied nanomaterials)	Reviews plant-derived probes and enzymatic methods for trace-level contaminant detection in soil. Proposes future AI-integrated arrays and CRISPR-based living sensors for scalable, predictive soil monitoring.
**36**	14	Nayak DS, 2023, INT J RECENT INNOV TRENDS COMPUT COMMUN	Not specified	Microbial biotechnology, IoT sensors	Artificial Intelligence (AI) for automation and regulation	Not specified	Reviews the integration of microbial biotechnology, IoT, and AI in vertical greenhouses for urban farming, showing improvements in crop yield (up to 40%) and reductions in water/fertilizer use (30%).

**37**	16	Rana S, 2022, BIOSENS NANOTHERANOSTICS	Antimicrobial resistance (AMR) genes	Not specified	AI-driven predictive management	Ozone nanobubbles, nanoparticles	Reviews biofilm control strategies in agricultural irrigation networks, proposing a management model combining real-time monitoring, passive design, and AI for sustainable solutions like phage therapy and ozone nanobubbles.
**38**	5	Duan C, 2021, FRONT MICROBIOL	Tetracycline hydrochloride, ofloxacin	Fluorescence sensing, MOF-based sensors	Machine Learning (Python-based RGB-ExponentialFit Analyzer)	Metal-Organic Frameworks (MOFs), nano-UiO-67	Developed an ML-assisted visual sensing platform (MOF-based) for simultaneous detection of tetracycline and ofloxacin in complex matrices (water, milk), enabling rapid, cost-effective, and portable analysis.
**39**	17	Asad M, 2025, ELECTROCHIM ACTA	Not specified	Enzyme-based, immunosensor, DNA/aptamer-based biosensors	Artificial Intelligence (AI), Microfluidics	Nanotechnology	Reviews the distinctive features and emerging applications of biosensors in healthcare, environmental monitoring, and food safety, highlighting the role of AI, microfluidics, and wearable devices.
**40**	12	Gupta YD, 2024, ARTIF INTELL MACH LEARN DRUG DES DEV	Not specified	Not specified	Machine Learning (supervised learning, transfer learning, Bayesian optimization)	Powder-based functional materials (carbon nanomaterials, metallic nanoparticles, MOFs/COFs)	Reviews AI-guided design of powder-based nanomaterials for smart textiles, enabling energy harvesting, sensing, and actuation. Discusses challenges like data scarcity and sustainability.
**41**	9	Sen D, 2024, GENE GENOME EDIT	Fusarium mycotoxins (fumonisin B1), VOCs	Electrochemical, optical, plasmonic biosensors	Machine Learning algorithms	Nanocarriers, stimuli-responsive nanomaterials	Proposes Fusarium-responsive smart materials that detect fungal pathogens via biochemical signatures and deliver on-demand antifungals, with AI-driven sensor arrays for enhanced field robustness.

**42**	18	Pandey RP, 2025, CRC PRESS	Air pollutants (benzene, SO2, NO2, BaP), CA4, CAT, SPP1, CDH1, TIMP1	Transcriptomics (WGCNA), Network Toxicology, Molecular Docking	Artificial Intelligence-driven pattern recognition, Machine Learning	Not specified	Identified 5 hub genes linking air pollution to lung adenocarcinoma. Machine learning and molecular docking validated high-affinity binding between benzo[a]pyrene and the CA4 protein, suggesting a mechanistic pathway.
**43**	45	Alexa M, 2025, BIORXIV	Micro- and nanoplastics (PE, PP, PS, PET, PVC)	μ-Raman, LDIR, μ-FTIR, Py-GC/MS, human biomonitoring	Not specified	Not specified	Quantitative analysis of 70 human biomonitoring studies for micro- and nanoplastics, providing exposure profiles and identifying critical knowledge gaps in analytical standardization and health risk assessment.
**44**	19	Kumari R, 2025, CHEMPHYSCHEM	Pesticides (nicosulfuron, 2,4-D, chlorpyrifos, cypermethrin) and their metabolites	Nanozyme sensor array (10-channel)	Machine Learning (Bayesian-optimized Random Forest - BO-RF)	Cu-carboxylate nanozymes	Developed an ML-assisted multi-channel nanozyme sensor array for simultaneous identification and metabolic analysis of multiple pesticides, achieving >97% prediction accuracy across different metabolic stages.
**45**	46	Wu R, 2024, CURR OPIN BIOTECHNOL	ctDNA, CTCs, miRNAs	Optical, electrochemical, magnetic, piezoelectric biosensors	AI for device design, signal processing, and clinical workflow integration	Carbon-based NPs, metallic frameworks, polymeric composites	Reviews cutting-edge nanobiosensors for cancer diagnosis and precision medicine, highlighting how AI enhances sensor performance and how nanomaterials enable multifunctional platforms for detection and therapy.
**46**	11	Getahun YA, 2022, VET MED RES REP	Not specified	Capacitive, resistive, piezoelectric, triboelectric, optical sensing	Artificial Intelligence (AI) integration	Conductive polymers (PEDOT:PSS), carbon nanomaterials (graphene), piezoelectric materials (ZnO)	Reviews e-textiles for real-time monitoring of health markers, energy storage, and therapeutic delivery. Highlights hybrid materials, multimodal sensing, and future integration with AI and biodegradable materials.

**47**	35	Jiang J, 2025, GENOME MED	Not specified	Microwave-assisted waste valorization (MWV), techno-economic analysis, life-cycle analysis	AI-driven optimization	Carbon nanomaterials (CNTs, CNFs, graphene-like structures)	Reviews microwave-assisted conversion of solid waste to hydrogen and carbon nanomaterials. Shows AI and renewable power integration can optimize yield, achieving >60% H2 concentration and significant energy savings.
**48**	37	Ali M, 2023, INT J EDUC MATH SCI TECHNOL	Not specified	Not specified	Artificial Intelligence (AI)-assisted agricultural management	2D materials (graphene, MXenes, TMDs, nanographene oxide), nanowires	Discusses the principles and applications of 2D material-based biosensors for precision agriculture (soil/crop monitoring), highlighting the role of AI for future scalable sensor networks and smart farming.
**49**	21	Khan A, 2022, FINANCE RES LETT	Zolpidem (ZLP) and its metabolites	Chromatography (HPLC, LC-MS/MS, UHPLC-MS/MS), spectrophotometry, electrochemistry	Artificial Intelligence (AI), Machine Learning (ML), Lab-on-a-chip (LOC)	Nanomaterials, molecularly imprinted polymers (MIPs)	Comprehensive review of analytical strategies for zolpidem, highlighting the potential of AI, ML, and LOC for automation, real-time monitoring, and predictive analysis in pharmaceutical and environmental samples.
**50**	10	Vitale M, 2023, ANTIBIOTICS	Ammonia, salinity, pathogens	Electrochemical biosensors	AI-based biosensing	Not specified	Reviews the application of biosensors in aquaculture for detecting environmental changes (ammonia, salinity), disease outbreaks, and stress factors, highlighting the role of modern technologies for timely intervention.
**51**	23	Ramos Cordeiro E, 2024, J KNOWL MANAG	Not specified	Computational modeling (deep learning)	Deep Learning (reproducibility study), CodeCarbon for emission measurement	Not specified	A reproducibility study of a deep learning model (PiDeeL), highlighting challenges in replicating computational experiments and calling for better documentation and algorithmic peer review for model reusability.

**52**	29	Decouttere C, 2021, GLOBAL HEALTH	Not specified	CRISPR/Cas9, Genomic Selection (GS), Environmental GS, Multi-omics (transcriptomics, proteomics, metabolomics, epigenomics)	Artificial Intelligence (AI), High-throughput phenotyping	Nanoparticles (for gene delivery)	Reviews biotechnological strategies to enhance maize resilience to climate change (drought, heat). Emphasizes integration of multi-omics, genome editing, AI, and nanoparticle-based gene delivery.
**53**	34	Olatunji AO, 2024, ENG SCI TECHNOL J	Not specified	Genomics, transcriptomics, proteomics, metabolomics	Machine Learning, Metabolic modelling	Not specified	Reviews the use of integrative multi-omics and bioinformatics (e.g., DESeq2) to understand microbial bioleaching mechanisms, highlighting how ML can predict interactions and enhance consortia for metal recovery.
**54**	30	Moreno-Indias I, 2021, FRONT MICROBIOL	Not specified	Electrochemical, optical, piezoelectric biosensors, CRISPR-based biosensors, microfluidics	Artificial Intelligence (AI), IoT	Nanomaterials	Reviews advances in biosensing technology for plant pathogen detection, emphasizing the potential of AI, IoT, and digital agriculture for predictive diagnostics and precision crop management.
**55**	36	Wan F, 2021, PHARMACEUTICS	Heavy metals	Biosensors (enzymes, DNAzymes, aptamers)	Machine Learning (Random Forest, Gradient Boosting, Neural Networks)	Not specified	Used ML algorithms (Random Forest best) to optimize biosensor parameters for trace heavy metal detection in water, significantly improving predictive accuracy for limit of detection and linearity.
**56**	22	Behl A, 2022, TECHNOL FORECAST SOC CHANGE	Phytochemicals	Supercritical fluid extraction, ultrasound/microwave/enzyme-assisted extraction,	AI-driven optimization, smart sensors	Nanomaterials (implied)	Discusses future trends in botanical extract development, including green technologies, AI for optimization, and multi-omics (metabolomics) for quality control, safety assessment, and discovery of bioactive compounds.

				metabolomics, chemometrics			
**57**	43	Chen C, 2025, ENVIRON SCI TECHNOL	Tetracycline	High-resolution mass spectrometry, Density Functional Theory (DFT) calculations	Machine Learning (ML)	Not specified	Developed an ML model informed by DFT to predict Gibbs free energy changes in photocatalytic antibiotic degradation. This framework maps reaction networks and assesses ecological risks of transformation products.
**58**	32	Buelow E, 2020, WATER RES X	Organic co-contaminants	Metagenomics, culturomics, high-throughput screening, genome-scale metabolic modeling	Artificial Intelligence (AI)/Machine Learning (ML)	Not specified	Discusses using synthetic microbiomes (SynMicro) and advanced techniques (metagenomics, AI/ML) to restore soil health under organic co-contamination, aiming for enhanced pollutant reduction and improved fertility.
